# COVID-19 Infection as a Possible Trigger for POLG-Related Mitochondrial Disease: A Case Report

**DOI:** 10.7759/cureus.77592

**Published:** 2025-01-17

**Authors:** Stanislava Suroviaková, Vladimir Zolak, Matúš Igaz, Jana Kršiaková, Peter Bánovčin

**Affiliations:** 1 Department of Pediatrics, Jessenius Faculty of Medicine and University Hospital, Martin, SVK; 2 Clinic of Genetic Medicine, Jessenius Faculty of Medicine and University Hospital, Martin, SVK

**Keywords:** alpers-huttenlocher syndrome, covid-19 infection, epilepsy, mitochondrial disorders, polg mutation

## Abstract

A six-year-old child presented with an acute onset of refractory epileptic seizures during a coronavirus disease 2019 (COVID-19) infection. As her clinical condition progressed, she developed super-refractory status epilepticus, resulting in significant cognitive and motor impairments. Genetic analysis revealed a homozygous mutation in the DNA Polymerase Gamma, Catalytic Subunit (*POLG)* gene (c.1399G>A; p.Ala467Thr), confirming a diagnosis of Alpers-Huttenlocher syndrome. The clinical course was characterized by refractory seizures and developmental regression, and it ultimately culminated in liver failure and multiorgan dysfunction, resulting in death. This case underscores the critical importance of early genetic evaluation in children with unexplained refractory seizures, particularly for detecting underlying mitochondrial disorders such as *POLG*-related syndromes. Mitochondrial function is highly sensitive to physiological and environmental stressors, including viral infections. Pathogens such as hepatitis viruses, influenza virus, HIV, respiratory syncytial virus (RSV), and severe acute respiratory syndrome coronavirus 2 (SARS-CoV-2) can exacerbate mitochondrial dysfunction. Therefore, identifying genetic vulnerabilities in these patients is essential for optimizing management strategies and potentially mitigating rapid clinical decline.

## Introduction

Mitochondria, often referred to as the powerhouses of eukaryotic cells, are essential for producing cellular energy through the oxidative phosphorylation (OXPHOS) system, which generates adenosine triphosphate (ATP). This intricate process is managed by the mitochondrial respiratory chain (MRC) in the inner mitochondrial membrane. Uniquely, mitochondria contain their own genome - mitochondrial DNA (mtDNA) that encodes key MRC protein subunits, while the remaining components, including approximately 1500 mitochondrial polypetides, are encoded by the nuclear genome (nDNA) [1,2]. The DNA Polymerase Gamma, Catalytic Subunit (*POLG)* gene, located on nuclear chromosome 15q, encodes the catalytic subunit of DNA polymerase gamma (pol γ), essential for mtDNA replication and repair. Mutations in the *POLG* gene are the most common causes of inherited mitochondrial disorders, affecting up to 2% of the population and resulting in a broad spectrum of mitochondrial diseases [3,4]. These mutations, with over 300 documented mutations in the *POLG* gene, known as *POLG* syndromes, exhibit significant clinical variability in onset, inheritance, and organ involvement [5]. Mitochondria are sensitive organels for infection, inflammation, or environmental insults that impact structural changes in mitochondrial membranes, and protein expression, resulting in their dysfunction [1,6].

There is increasing evidence that various viruses, including hepatitis viruses, influenza, human immunodeficiency virus (HIV), respiratory syncytial virus (RSV), and severe acute respiratory syndrome coronavirus 2 (SARS-CoV-2), can induce mitochondrial dysfunction [6,7]. Viruses can alter mitochondrial dynamics, including processes such as autophagy, OXPHOS, and enzymatic function. Additionally, they can reduce ATP production in mitochondria, diverting energy resources to support viral replication. This metabolic disruption induces cellular stress and mitochondrial damage, primarily through the generation of reactive oxygen species (ROS) [8]. The interaction between viral infections, mitochondrial disorders, and associated neurological outcomes has become an increasingly important area of research, especially in the context of the COVID-19 pandemic. SARS-CoV-2, specifically, has been shown to impair mitochondrial function via multiple mechanisms, such as the disruption of mitochondrial dynamics by increasing membrane permeability, hijacking of metabolic pathways, inhibition of mitochondrial antiviral signaling (MAVS), immune dysregulation, and induction of apoptosis [8-10]. Recent findings also suggest that viral infections, including COVID-19, may serve as triggers or accelerators for the onset or progression of mitochondrial diseases in genetically predisposed individuals [11].

We present the case of a six-year-old girl who developed super-refractory status epilepticus following a COVID-19 infection, which led to the diagnosis of a previously unrecognized *POLG*-related mitochondrial disorder. Despite intensive therapeutic interventions, her condition deteriorated rapidly, culminating in multiorgan failure and death. This case underscores the potential role of viral infections, particularly SARS-CoV-2, in exacerbating mitochondrial dysfunction and highlights the critical importance of early genetic testing in children presenting with unexplained refractory seizures.

## Case presentation

A six-year-old girl was admitted to our Pediatric Department with an acute onset of focal seizures. Several hours prior, after waking up in the morning, she experienced nausea, repeated vomiting, headaches, and lethargy, which were followed by the onset of seizures characterized by bulbar deviation, head turning to the right, impaired consciousness and focal jerking of the right hand. Initial anti-seizure medications (ASM) were ineffective, and despite receiving repeated boluses of intravenous benzodiazepines and loading doses of phenobarbital and valproic acid (VPA), the seizure persisted. Consequently, she was intubated, induced into a thiopental coma, and transferred to the Pediatric Emergency Department in our hospital for further management of seizures. 

This patient was born to non-consanguineous Caucasian parents and has a healthy 16-year-old sister. She was the second child from a physiological, uncomplicated pregnancy, with no history of miscarriages. The family history was unremarkable, and her birth and developmental milestones were normal for her age. She had overcome common childhood illnesses, and her immunizations were up to date. At the age of three, she began experiencing episodic attacks characterized by sudden-onset ataxia, lethargy, and headache, accompanied by nausea and vomiting. These episodes occurred approximately once a month and lasted several hours. The patient was evaluated by a pediatric neurologist, whose physical examination revealed no pathological neurological findings. She underwent two magnetic resonance imaging (MRI) of the brain and an electroencephalogram (EEG), all of which had normal results. These episodes were ultimately diagnosed as migraine. 

Upon admission to our hospital, persistent focal myoclonic jerks were observed in the patient's right hand. The patient presented with a high-grade fever. The parents reported that several days prior to the onset of seizures, the child had a cough, rhinorrhea, and a sore throat, but no fever. Initial laboratory tests, including inflammatory markers, were within normal limits, with lactic acid, and aminotransferases also showing normal values. Blood analysis revealed a normal white blood cell count alongside neutrophilia, lymphocytopenia, and mild anemia (Table 1).

**Table 1:** Initial laboratory parameters, including normal ranges CRP = C-reactive protein; PCT = procalcitonin; WBC = white blood cells; Hgb = hemoglobin; AST = aspartate aminotransferase; ALT = alanine aminotransferase; GGT = gamma-glutamyl transferase.

Laboratory parameters	Result	Normal ranges
CRP (mg/dL)	2.80	< 5
PCT (ng/mL)	0.18	< 0,5
WBC (x 10^9^/L)	9.80	4 - 12
Platelets (x 10^9^/L)	347	150 - 450
Hgb (g/L)	106	110 - 150
Neutrophils (%)	81.2	45 - 60
Lymphocytes (%)	17.6	30 - 45
AST (ukat/L)	0.34	0.10 – 0.60
ALT (ukat/L)	0.40	0.10 – 0.60
GGT (ukat/L)	0.14	0.07 – 0.37
Lactic acid (mmol/L)	2.05	0.56 – 2.25
Ammonia (umol/L)	43.0	16.0 – 53.0

A chest X-ray revealed pneumonic consolidation on the right side. Although the initial reverse transcription polymerase chain reaction (RT-PCR) test for SARS-CoV-2 was negative, serological testing indicated the presence of positive levels of immunoglobulin (Ig)M and IgG antibodies against SARS-CoV-2. Consequently, the patient was initiated on empirical treatment with ceftriaxone and macrolides, in addition to symptomatic care and vitamin therapy. Follow-up chest X-rays conducted during the treatment showed no evidence of an inflammatory process.

Due to the persistent myoclonic jerks, the ASM regimen was escalated with the initiation of a continuous infusion of midazolam. This treatment, in conjunction with thiopental and valproic acid, led to the cessation of the seizures. The EEG displayed a delayed, burst suppression“ pattern, prompting a gradual reduction of general anesthesia after 24 hours. An initial brain computed tomography (CT) scan revealed no pathological findings. A lumbar puncture (LP) showed elevated protein levels, with no other abnormalities (Table 2).

**Table 2:** Initial cerebrospinal fluid results, including normal ranges CSF = cerebrospinal fluid

Lumbar puncture results	Result	Normal ranges
CSF Glucose (mmol/L)	3.0	1.8 – 4.6
CSF Protein (g/L)	1.03	0.1 – 0.45
CSF Neutrophils (x 10^6^/L)	0	< 1
CSF Lymphocytes (x 10^6^/L)	0	< 5
CSF Lactatic acid (mmol/L)	1.37	1.10 – 1.80

**Figure 1:**
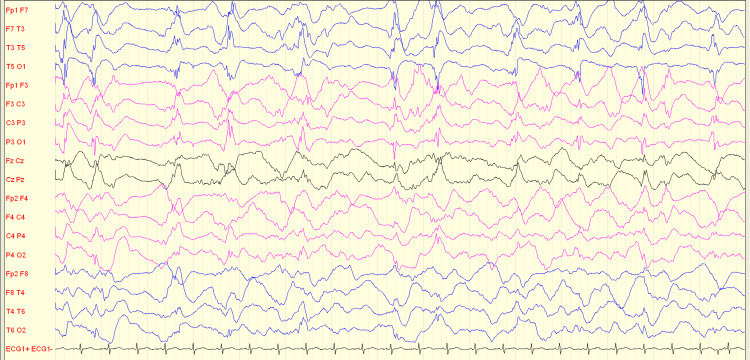
Section of scalp EEG with rhythmic high amplitude delta with superimposed spikes in left hemisphere (RHADS pattern) that is typical for AHS RHADS = rhythmic high-amplitude delta activity with superimposed spikes; AHS = Alpers-Huttenlocher syndrome.

**Figure 2:**
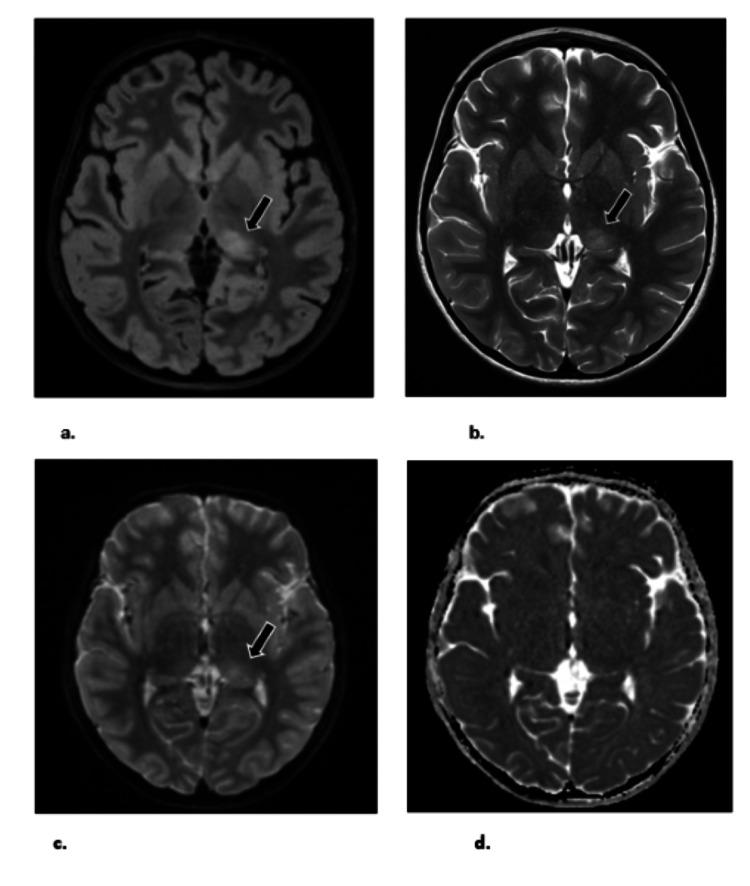
Brain MRI showed T2/FLAIR hyperintensity in left thalamus a. FLAIR, b. T2, c. DWI, d. ADC. FLAIR = fluid-attenuated inversion recovery; T2 = T2 weighted image MRI; DWI = diffusion-weighted imaging; ADC = apparent diffusion coefficient.

Autoantibodies associated with autoimmune encephalitis (AE) were not detected in cerebrospinal fluid (CSF) or serum. During the reduction of general anesthesia, the EEG revealed left-sided rhythmic high-amplitude delta waves superimposed with epileptiform discharges (Figure 1). A subsequent brain MRI showed remarkable hyperintensity in the left thalamus on T2-weighted-fluid-attenuated inversion recovery (T2/FLAIR) sequences, along with diffusion restriction (DWI), but without any changes in apparent diffusion coefficient (ADC) or abnormal enhancement (Figure 2). 

After completing general anesthesia, the patient developed epilepsia partialis continua (EPC), characterized by continuous right-sided acral myoclonic jerks in hand. Adjustments to the ASM regiment were necessary due to a slight increase in aminotransferases (aspartate aminotransferase (AST) 0.87 ukat/L, alanine aminotransferase (ALT) 0.63 ukat/L, gamma-glutamyl transferase(GGT) 0.17 ukat/L). After three days of treatment, VPA was discontinued and replaced with levetiracetam, subsequently introducing lacosamide, topiramate, and phenytoin. Despite negative antibody results for AE, we initiated empirical treatment with methylprednisolone, intravenous immunoglobulin (IVIG), and plasma exchange starting on the 12th day of hospitalization in response to the progression of the clinical condition. On the 21st day of hospitalization, a modified ASM regimen (phenytoin, topiramate, brivaracetam) achieved partial control of seizures. The patient's level of consciousness improved, allowing her to respond affirmatively or negatively to simple queries, although intermittent right-sided acral myoclonus persisted.

**Figure 3:**
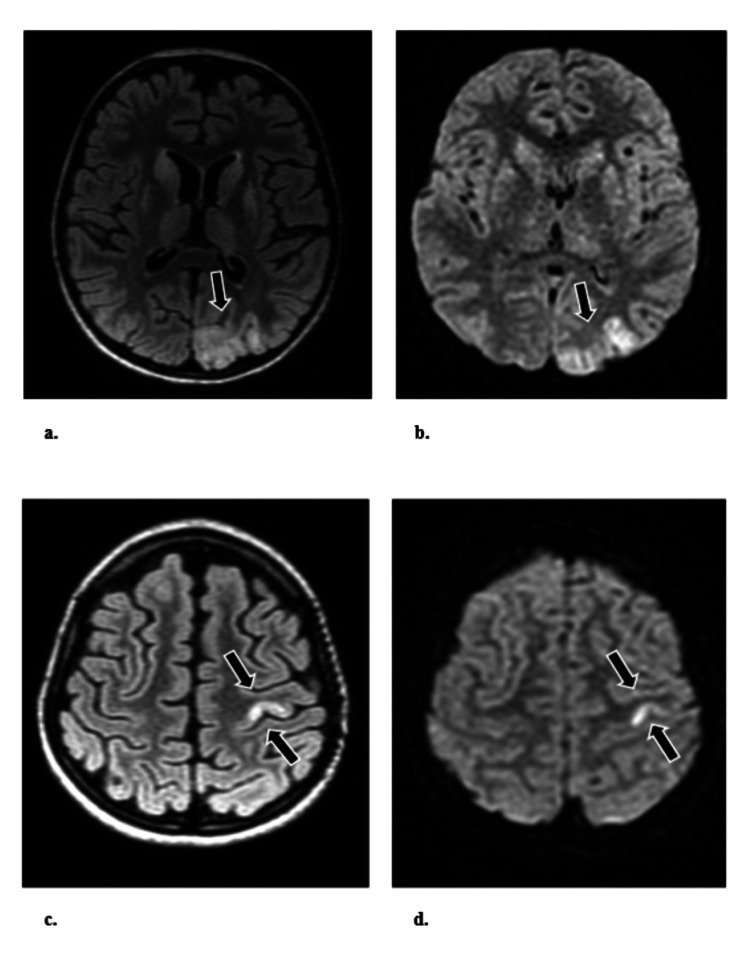
MRI brain (left occipital gray matter and left precentral gyrus) MRI brain showed multiple T2/FLAIR hyperintensities in the left occipital gray matter (a. FLAIR, b. DWI) and in the left precentral gyrus (c. FLAIR, d. DWI) that are typical for AHS. FLAIR = fluid-attenuated inversion recovery; T2 = T2 weighted image MRI; DWI = diffusion-weighted imaging; AHS = Alpers-Huttenlocher syndrome.

However, on the 38th day of hospitalization, the patient's clinical status deteriorated, manifesting EPC with right-sided lateralization and worsening consciousness. Cerebrospinal fluid (CSF) analysis revealed the progression of hyperproteinorrachia (protein 1.57 g/L). Serum analysis showed anemia (Hgb 87 g/L), and elevated aminotransferases (AST 1.2 ukat/L, ALT 0.6 ukat/L). Abdominal ultrasonography displayed normal findings. MRI revealed new multiple T2/FLAIR hyperintensities in the left occipital gray matter, bilateral basal ganglia, left precentral gyrus, and a stationary lesion in the left thalamus, along with an increase in the subarachnoidal spaces of the bilateral hemispheres (Figure 3).

In light of the clinical deterioration, despite the early initiation of immunotherapy and combined ASM, we began to consider other potential causes of super-refractory status epilepticus. Consequently, genetic testing was initiated, specifically a Next-Generation Sequencing (NGS) panel for genes associated with epilepsy.

**Figure 4:**
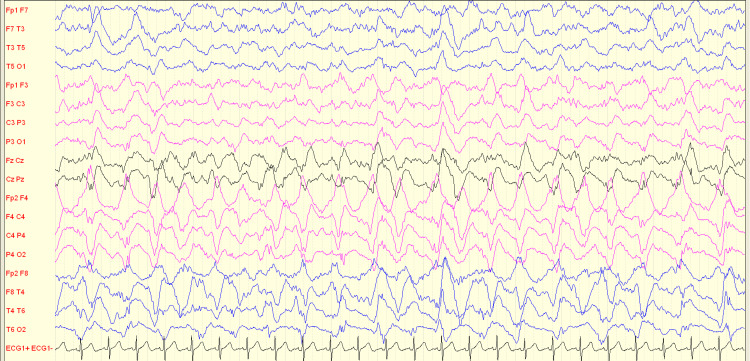
Section of scalp EEG with typical RHADS pattern in right hemisphere. EEG = electroencephalogram; RHADS = rhythmic high-amplitude delta activity with superimposed spikes.

Despite adjustments to the ASM regimen (phenytoin, clobazam, continuous midazolam), the child's clinical status continued to deteriorate. There was a deepening impairment of consciousness, persistent right-sided myoclonic jerks, and the emergence of jerks in the left hand. The EEG displayed continuous right-sided rhythmic delta activity with superimposed fast and sharp components (Figure 4).

Laboratory tests indicated a progression in aminotransferases levels (AST 2.8 ukat/L, ALT 0.8 ukat/L), and elevated serum lactate (2.9 mmol/L). On the 65th day of hospitalization, myoclonic jerks persisted throughout the body, necessitating the induction of a thiopental-induced coma. Unfortunately, the clinical condition progressed to multiorgan failure, ultimately resulting in an adverse outcome. The results of the genetic tests were obtained shortly after the patient's death and revealed a homozygous frameshift c.1399G>A p.(Ala467Thr) variant in exon 7 of *POLG* gene (NM_002693.3).

## Discussion

In this case study, a six-year-old girl presented with the acute onset of EPC in the context of a confirmed COVID-19 infection. As her clinical condition deteriorated, she progressed to super-refractory status epilepticus, ultimately leading to the diagnosis of an underlying *POLG*-related mitochondrial disease. This case shows the potential interaction between viral infections, particularly SARS-CoV-2, and mitochondrial diseases, highlighting the need for heightened vigilance in the management of pediatric patients presenting with unexplained refractory seizures.

In this patient, the identified pathogenic genetic variant c. 1399G>A (p.Ala467Thr) is the most prevalent pathogenic mutation in the *POLG* gene among the Caucasian population, occurring in 0.6% of certain populations. While many patients, including our case, are homozygous for this mutation, the clinical manifestation can vary significantly, ranging from childhood-onset Alpers-Huttenlocher syndrome (AHS) to adult-onset sensory ataxic neuropathy, dysarthria and ophthalmoparesis, or spinocerebellar ataxia with epilepsy [12]. In AHS, mutations can present as homozygous recessive or as compound heterozygous forms. Tzoulis et al. [13] identified a large cohort of patients with the p.A467T and p.W748S mutations in the *POLG* gene, who presented with a mitochondrial spinocerebellar ataxia and AHS. Homozygous recessive mutations typically result in a milder phenotype with a later onset, while compound heterozygous mutations are often associated with an earlier onset and a more severe, progressive disease course. However, exceptions have been documented wherein identical homozygous mutations can manifest as severe early-onset disease in one patient and a milder form in another. Similarly, identical compound heterozygous mutations can present variability, leading to either mild or severe phenotypes [5,13]. The variability in disease onset and severity in mitochondrial disorders remains incompletely understood, suggesting that clinical outcomes are modulated not only by mutation type and location but also by additional genetic, epigenetic, immunological, and environmental factors, including infections [5]. Saneto and Naviaux proposed a model of strong genotype-environment interactions in mitochondrial disease expression [14]. Specifically, in *POLG*-related disorders, genetic predispositions may remain clinically silent until activated by specific environmental triggers, such as exposure to particular medications, viral infections, or physiological stressors.

Recent data underscore the critical role of aberrant immune activation in the pathogenesis of certain genetic mitochondrial diseases, influencing both the onset and severity of symptoms [15]. Viral infections, in particular, are recognized as a major cause of morbidity and mortality in pediatric patients with mitochondrial disorders, frequently precipitating organ dysfunction that can lead to rapid, fatal outcomes. The risk is especially pronounced in children under six years of age due to their immature and often compromised immune systems [16]. For example, a study involving 14 pediatric patients with mitochondrial leukoencephalopathy in India found febrile infections to be the initiating event in 57% of cases [17], with the energic stress associated with immune response activation identified as a possible mechanism [15]. In individual case reports, infectious triggers have been noted to precipitate mitochondrial crises: for example, a three-year-old child with acute onset severe epilepsy was diagnosed with a POLG-related mitochondrial disorder following an episode of acute Lyme borreliosis [18]. Similarly, Al-Zubeidi et al. (2023) reported HHV-6 encephalitis as a precipitant of underlying mitochondrial dysfunction [19]. Supporting these findings, in a multicenter European study of 130 patients with Leigh syndrome, infections were implicated in 61% of acute exacerbations leading to hospitalization [20], underscoring the pivotal role of infections in exacerbating the progression of mitochondrial diseases.

We propose that in our patient, SARS-CoV-2 infection may have been the critical trigger precipitating super-refractory status epilepticus, likely exacerbating underlying mitochondrial dysfunction. Typically, infants and children with AHS appear healthy until disease onset, although some may exhibit non-specific developmental delays [21]. The progression and clinical deterioration of mitochondrial disorders are often associated with intercurrent infection [21,22]. While infections do not directly cause AHS, they can reveal or trigger the disease in previously asymptomatic children or young adults with *POLG* mutations, thereby accelerating the stepwise progression of the disorder. Notably, a series of cases indicated that 12 out of 15 children with confirmed *POLG* mutations presented their initial symptoms within 3-10 days following an intercurrent infection [5,23]. Viral infections, more so than bacterial, tend to exacerbate AHS, likely due to the role of mitochondria in innate immunity [5]. Mitochondria play a central role in antiviral defense, and disruptions by viral pathogens can further compromise mitochondrial function [6,7], thereby accelerating disease progression in individuals with underlying mitochondrial disorders such as AHS.

AHS is classified as a mitochondrial DNA (mtDNA) depletion syndrome. Although over 70% of patients have normal mtDNA copy numbers in the liver and muscle at symptom onset, progressive depletion becomes evident as the disease advances. In later stages, mtDNA levels can drop to less than 35% of normal in critical tissues, such as the liver, brain, and muscle [5]. It is hypothesized that the excessive oxidative stress and mitochondrial dysfunction induced by SARS-CoV-2 could further accelerate mtDNA depletion and hasten the progression of AHS. While speculative, the role of COVID-19 in exacerbating this patient's condition is plausible, given its known association with neurological complications and mitochondrial dysfunction. A recent report described three patients who developed mitochondrial encephalomyopathy, lactic acidosis, and stroke-like episodes (MELAS) syndrome following COVID-19 infection, suggesting that the virus may act as a trigger for mitochondrial disorders [11].

In the initial therapeutic management of the child's seizures, VPA was administered, which may have contributed to the progression of AHS, particularly in the context of a coincidental COVID-19 infection. Due to a mild elevation in aminotransferase levels, VPA was discontinued after three days, leading to the normalization of liver enzyme levels. VPA is known to precipitate liver failure in individuals with AHS, though it is important to note that some patients may develop liver failure even without prior exposure to VPA [3]. Additionally, VPA-induced hepatotoxicity can occasionally be reversible. In a reported case, a two-year-old boy with AHS recovered normal liver function after discontinuing VPA, and the diagnosis of AHS was confirmed thereafter [24.

## Conclusions

This case underscores the significant interaction between viral infections, such as SARS-CoV-2, and genetic predisposition to mitochondrial dysfunction in pediatric patients with refractory seizures. The rapid deterioration of disease in our patient with previously undiagnosed *POLG*-related mitochondrial disorders suggests that SARS-CoV-2 induced oxidative stress and mitochondrial impairment accelerated mtDNA depletion, leading to super-refractory status epilepticus, multiorgan failure, and a fatal outcome. The presence of the homozygous c.1399G>A (p.Ala467Thr) mutation in the *POLG* gene, commonly associated with AHS, likely exacerbated the mitochondrial dysfunction triggered by SARS-CoV-2. Recognizing the interactions between genotype and environmental factors, such as infections, is essential for understanding the mechanisms underlying POLG-related diseases and improving therapeutic strategies.

This case also emphasizes the essential role of early genetic testing in guiding appropriate clinical management and identifying patients at heightened risk for adverse outcomes when exposed to specific medications or environmental stressors, such as viral infections.

## References

[1] Osellame LD, Blacker TS, Duchen MR (2012). Cellular and molecular mechanisms of mitochondrial function. Best Pract Res Clin Endocrinol Metab.

[2] Viscomi C, Zeviani M (2017). MtDNA-maintenance defects: syndromes and genes. J Inherit Metab Dis.

[3] Rahman S, Copeland WC (2019). POLG-related disorders and their neurological manifestations. Nat Rev Neurol.

[4] Barca E, Long Y, Cooley V (2020). Mitochondrial diseases in North America: an analysis of the NAMDC registry. Neurol Genet.

[5] Saneto RP (2016). Alpers-Huttenlocher syndrome: the role of a multidisciplinary health care team. J Multidiscip Healthc.

[6] Elesela S, Lukacs NW (2021). Role of mitochondria in viral infections. Life (Basel).

[7] Sun Z, Wang Y, Jin X, Li S, Qiu HJ (2024). Crosstalk between dysfunctional mitochondria and proinflammatory responses during viral infections. Int J Mol Sci.

[8] Singh SP, Amar S, Gehlot P, Patra SK, Kanwar N, Kanwal A (2021). Mitochondrial modulations, autophagy pathways shifts in viral infections: consequences of COVID-19. Int J Mol Sci.

[9] Targhetta VP, Amaral MA, Camara NO (2021). Through DNA sensors and hidden mitochondrial effects of SARS-CoV-2. J Venom Anim Toxins Incl Trop Dis.

[10] Jesenak M, Brndiarova M, Urbancikova I (2020). Immune parameters and COVID-19 infection - associations with clinical severity and disease prognosis. Front Cell Infect Microbiol.

[11] Ramezani M, Rabiei MM, Cheraghi Z, Simani L (2023). The possible role of COVID-19 in the triggering of underlying mitochondrial dysfunction in MELAS syndrome, a brief report of three cases. Acta Neurol Taiwan.

[12] Neeve VC, Samuels DC, Bindoff LA (2012). What is influencing the phenotype of the common homozygous polymerase-γ mutation p.Ala467Thr?. Brain.

[13] Tzoulis C, Engelsen BA, Telstad W (2006). The spectrum of clinical disease caused by the A467T and W748S POLG mutations: a study of 26 cases. Brain.

[14] Saneto RP, Naviaux RK (2010). Polymerase gamma disease through the ages. Dev Disabil Res Rev.

[15] Hanaford A, Johnson SC (2022). The immune system as a driver of mitochondrial disease pathogenesis: a review of evidence. Orphanet J Rare Dis.

[16] Eom S, Lee HN, Lee S, Kang HC, Lee JS, Kim HD, Lee YM (2017). Cause of death in children with mitochondrial diseases. Pediatr Neurol.

[17] Bindu PS, Sonam K, Chiplunkar S (2018). Mitochondrial leukoencephalopathies: a border zone between acquired and inherited white matter disorders in children?. Mult Scler Relat Disord.

[18] Gaudó P, Emperador S, Garrido-Pérez N (2020). Infectious stress triggers a POLG-related mitochondrial disease. Neurogenetics.

[19] Al-Zubeidi D, Thangarajh M, Pathak S (2014). Fatal human herpesvirus 6-associated encephalitis in two boys with underlying POLG mitochondrial disorders. Pediatr Neurol.

[20] Sofou K, De Coo IF, Isohanni P (2014). A multicenter study on Leigh syndrome: disease course and predictors of survival. Orphanet J Rare Dis.

[21] Saneto RP, Cohen BH, Copeland WC, Naviaux RK (2013). Alpers-Huttenlocher syndrome. Pediatr Neurol.

[22] Naviaux RK (2014). Metabolic features of the cell danger response. Mitochondrion.

[23] Nguyen KV, Østergaard E, Ravn SH (2005). POLG mutations in Alpers syndrome. Neurology.

[24] McFarland R, Hudson G, Taylor RW (2008). Reversible valproate hepatotoxicity due to mutations in mitochondrial DNA polymerase gamma (POLG1). Arch Dis Child.

